# Bilateral Synchronous Stress Fracture of the Tibia in a Young Female Basketball Player

**DOI:** 10.5334/jbr-btr.1042

**Published:** 2016-05-16

**Authors:** Magdalena Posadzy, Filip Vanhoenacker

**Affiliations:** 1 Orthopaedic and Rehabilitation University Hospital K.Marcinkowski University of Medical Sciences in Poznan, Poland, PL; 2 AZ Sint-Maarten and University (Hospital) Antwerp/Ghent, BE

**Keywords:** Knee pain in athletes, Bilateral stress fracture, Plain radiography, MRI

## Abstract

We present a case of a bilateral synchronous stress fracture of the tibia in a young female basketball player. The patient was initially referred for ultrasound and radiographs of the knees to exclude Osgood-Schlatter disease. Radiographs and subsequent MRI revealed bilateral stress fractures of the proximal tibia. A synchronous and symmetrical occurrence of stress fractures in the lower limbs is unusual. As clinical presentation is often nonspecific, appropriate imaging (plain films and MRI) plays a pivotal role in the correct diagnosis of this uncommon entity.

## Case Presentation

An 11-year-old female basketball player was referred to our radiology department with anterolateral pain of both knees over a few weeks to exclude Osgood-Schlatter disease. Previous medical history consisted of Perthes disease of the right hip at the age of 4.

**Figure 1:**
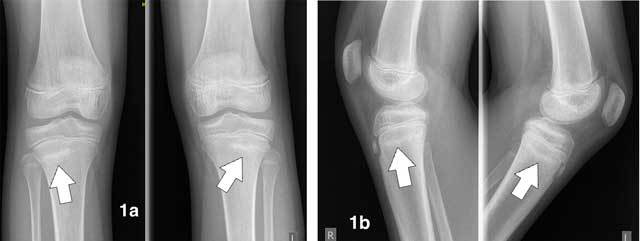
Plain radiographs of knees. **a.** Anteroposterior view of the right and left knee. **b.** lateral view of the right and left knee. Note sclerotic lines parallel to the growth plates at the lateral aspect of both proximal tibiae (white arrows) in keeping with synchronous stress fractures.

**Figure 2:**
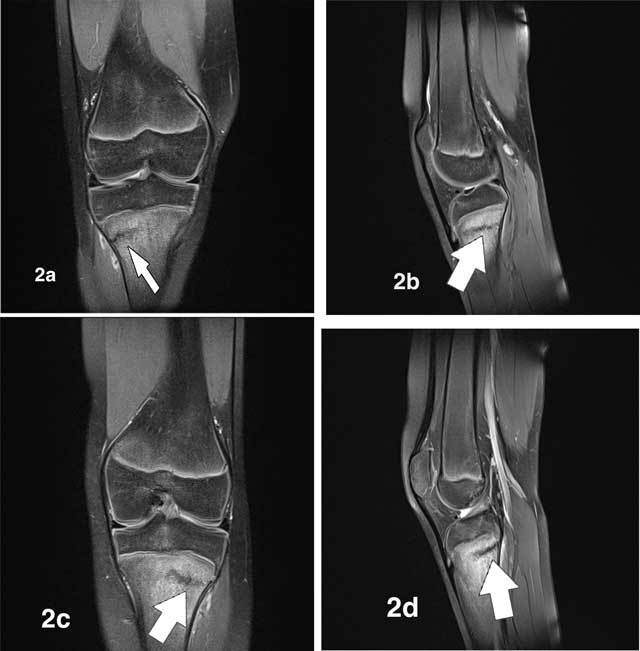
On fatsuppressed (FS) T2-weighted fat-saturated MR images, the fracture lines (white arrows) are of low signal and are surrounded by an extensive high-signal area of bone marrow oedema. **a.** Coronal FS T2-weighted image (WI) of the right knee. **b.** Sagittal FS T2-WI of the right knee. **c.** Coronal FS T2-WI of the left knee. **d.** Sagittal FS T2-WI of the left knee.

**Figure 3:**
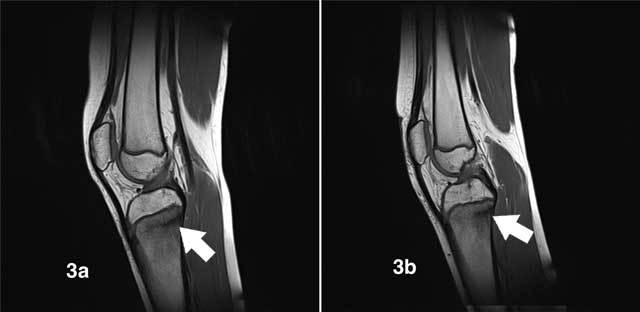
On T1-weighted images, the fracture lines are of low signal (white arrows) and are surrounded by bone marrow edema, which is of relatively low signal compared to normal bone marrow. **a.** Sagittal T1-WI of the right knee. **b.** Sagittal T1-WI of the left knee.

Ultrasound (US) revealed bilateral normal appearance of the tibial tubercle, excluding Osgood-Schlatter disease. Subsequent conventional radiographs of both knees showed sclerotic lines parallel to the growth plates in keeping with synchronous stress fractures of both proximal tibiae (Figures 1a, 1b). Additional MRI depicted low-intensity fracture lines surrounded by bone marrow oedema, confirming the diagnosis of stress fractures (Figures 2a, 2b, 2c, 2d, 3a, 3b). On T2-weighted fat-saturated images we observed an extensive high-signal area of bone marrow oedema surrounding fracture lines (Figures 2a, 2b, 2c, 2d). On T1-weighted images, the oedematous marrow changes have low signal intensity (Figures 3a, 3b).

Although the conventional radiography in our case was sufficient to allow for the diagnosis of stress fracture, MRI was performed to evaluate the precise extent of the fracture line and surrounding oedema and to exclude any underlying bone marrow disease. Laboratory examination was within normal limits. The patient was treated conservatively by rest. The recovery was uneventful, and the patient was completely pain free after four weeks.

## Discussion

Stress fractures most commonly affect athletes and dancers and occur after weight-bearing exercises which provoke repetitive stress to pelvis and lower limb bones, frequently the tibia, metatarsals, fibula, tarsal, and navicular bones. In sports medicine, those fractures account for over 10 percent of all injuries, but in runners, the incidence is even up to 31 percent of all injuries, with female predominance [1]. However, they are uncommonly diagnosed bilaterally at the same time. A discomfort during physical activity can be the initial manifestation, evolving to constant pain at rest.

Stress fracture is a condition when submaximal repetitive stress is applied on a healthy bone or normal forces targeted to weakened bone secondary to underlying disease. Most frequently, those conditions include disorders of calcium metabolism, inflammatory conditions (rheumatoid arthritis), nutrition deficiency, steroid use, or genetic factors. Dealing with bilateral stress fractures in the young population can be misleading and require alertness of systemic disease, including metabolic bone diseases, Still’s disease, anorexia nervosa, or a malignancy process, for example, leukemia. Frequently in athletes, stress fractures are a result of recent changes in training schedule in a short time span, both including increased intensity or duration [2,3].

Synchronous stress fractures are very rare [4]. Bilateral symmetrical localization, as in our case, is believed to result from symmetrical overload to the weakest parts of young bones, which biomechanically is the compression side most commonly situated in posterior aspect of the tibial diaphysis [3]. It should be emphasized, however, that the location of the stress fractures may differ with the age, the (sports) activity, and individual biomechanics [4]. Congenital or acquired bone deformities such as knock-knee or bowed legs can be predisposing factors, but in this case, they were excluded. In our patient, the fractures lines were located posterolaterally at the proximal tibiae.

Ultrasound is regarded as the initial imaging technique for assessment of knee pain in children and adolescents, particularly because of radiation restraints. Although ultrasound is excellent for evaluation of periarticular structures such as tendons and ligaments, it is far less sensitive for detection of stress fractures. Some authors reported the use of ultrasound in the diagnosis of stress fractures [5], but this cannot be recommended as the preferred technique in general. Targeted US on the area of focal pain may allow it to depict subtle changes of the external cortical bony outline, periosteum thickening, and increased local vascularity with color Doppler [2,6]. Although ultrasound is often used as a primary imaging modality to evaluate knee pain in children, one should not hesitate to perform additional plain films or MRI if pain persists or remains unexplained.

On conventional radiography, stress fractures typically present as band-like sclerotic areas, but in early stages, plain films may be normal. Even up to 85 percent of them can be missed on initial radiographs and about 50 percent on follow-up [7]. A triple-phase bone scan is very sensitive tool for this lesion, but the specificity is relatively low. The method of choice for diagnosis and staging of stress fractures is MRI. It shows the whole range of different grades of stress reactions, starting from periosteal oedema (grade 1), progressive periosteal and bone marrow oedema (grades 2–3), and finally cortical stress fracture (grade 4) [5,8].

Other causes of bilateral chronic onset pain at the knees and lower legs in young patients include middle tibial stress syndrome (MTSS), commonly designated as “shin splints”; chronic compartment syndrome (CCS); delayed onset muscle soreness (DOMS); and Osgood-Schlatter disease and more common causes of anterior knee pain (chondromalacia and patellofemoral maltracking).

The treatment of bilateral stress fractures of the proximal tibia is not different from unilateral stress fractures of the tibia and consists of rest or protected load bearing of the limbs, followed by modified activity and the graded return to a training schedule commensurate with bone healing [1].

The main complication includes progression to complete fracture, which is rare in cases of appropriate patient management.
